# Acclimation During the 7-Day CO-Synch + CIDR Protocol Improves Temperament and Pregnancy Rate to Timed Artificial Insemination in *Bos taurus* Beef Heifers

**DOI:** 10.3390/ani16131953

**Published:** 2026-06-24

**Authors:** Sydney Flax, Danielle M. Ellinghuysen, Allen G. Schwartz, Jack Lemmon, Joao V. C. Silva, Santiago P. Hurtado, Andreia Ferreira Machado, Victor E. Gomez-Leon, John R. Jaeger, Nicola Oosthuizen, Kenneth C. Olson, Felipe A. C. C. Silva, Sandy K. Johnson, Nicholas W. Dias

**Affiliations:** 1 Department of Animal Sciences and Industry, Kansas State University, Manhattan, KS 66506, USA; sydneyflax@ksu.edu (S.F.); dmstock@ksu.edu (D.M.E.); allenschwartz22@ksu.edu (A.G.S.); jacklemmon@ksu.edu (J.L.); joaaoovitto@gmail.com (J.V.C.S.); santiago.paez@altagenetics.com (S.P.H.); andreia_fmachado@hotmail.com (A.F.M.); vgomezleon@ksu.edu (V.E.G.-L.); kcolson@ksu.edu (K.C.O.); 2 Department of Animal Science, Universidade Federal de Viçosa, Viçosa, Minas Gerais 36570-900, Brazil; 3 Department of Animal Sciences and Industry, Kansas State University, Hays, KS 67601, USA; jrjaeger@ksu.edu; 4 Department of Animal Science, South Dakota State University, Brookings, SD 57007, USA; 5 ABS Global, DeForest, WI 53532, USA; nicola.oosthuizen@genusplc.com; 6 Department of Animal Science, North Carolina State University, Raleigh, NC 27695, USA; falvesc@ncsu.edu; 7 Northwest Research and Extension Center, Kansas State University, Colby, KS 67701, USA; sandyj@ksu.edu

**Keywords:** animal welfare, chute score, exit velocity, cortisol, low-stress handling, habituation, reproductive efficiency, replacement heifer

## Abstract

Temperament has been associated with reproductive success in beef cattle, with excitable animals often exhibiting reduced fertility. This study evaluated whether acclimating heifers to human handling during an ovulation synchronization protocol improves temperament and pregnancy rates to timed artificial insemination (TAI). A total of 622 *Bos taurus* yearling beef heifers across five locations and two breeding seasons (eight herd-year observations) were stratified according to reproductive maturity and temperament and were assigned to either acclimation (TRT; n = 307) or control (CTRL; n = 315). Acclimated heifers were moved through handling facilities without restraint prior to each protocol event (days 0, 7, and 10). Temperament was assessed using chute score (CS) and exit velocity (EV), and plasma cortisol was measured in a subset of animals. Acclimated heifers had lower CS on days 7 and 10 (*p* = 0.011 and *p* = 0.010, respectively) and greater pregnancy rates to TAI compared with control heifers (54.5% vs. 45.2%; *p* = 0.018). Exit velocity and cortisol concentrations did not differ between treatments (*p* ≥ 0.13). These results indicate that acclimation during handling events can improve behavioral responses and pregnancy rates to TAI with modest additional handling time (a mean of 17 s per heifer; no more than 18 min per location/day), providing a practical and scalable strategy for beef producers.

## Simple Summary

Cattle temperament is defined as the magnitude of excitability and stress response an individual will experience when handled, with excitable heifers showing decreased fertility when handled for reproductive protocols. This study evaluated whether acclimating beef heifers to human handling and facilities during routine reproductive protocol handling events could improve their behavior and reproductive performance. A total of 622 heifers were either acclimated by being moved through the handling facility without restraint prior to each protocol event or managed conventionally. Acclimated heifers exhibited calmer behavior during handling and achieved greater pregnancy rates to timed artificial insemination. The acclimation procedure required only modest additional handling time (about 17 s per heifer). These findings suggest that a simple, time-efficient acclimation strategy can improve both animal welfare and reproductive success in beef production systems.

## 1. Introduction

To improve the reproductive efficiency of cow–calf operations, it is key to maximize the number of females that become pregnant in a timely manner. The goal of producing one calf per cow every year has thus become a standard considered vital for the efficiency of production and profitability. Achieving this goal requires strategic management of heifer development to optimize their reproductive success in their first year. In essence, the success or failure of a heifer to become pregnant early in her first breeding season affects her performance in subsequent years [1]. Furthermore, heifers that calve early in their first calving season have greater longevity and wean heavier calves than those that calve later [2,3]. Timing of first parturition can thus influence heifer productivity for up to six subsequent calving seasons, ultimately determining her ability to produce one calf per year. Therefore, becoming pregnant early in her first breeding season is of great importance. An effective management strategy to increase the number of heifers that become pregnant early in the breeding season is to implement an ovulation synchronization (OS) protocol and artificial insemination (AI) or timed artificial insemination (TAI) [4].

The success of OS and TAI is influenced by many factors, including heifer temperament. Cattle temperament is defined as the behavioral response to human handling [5], and previous studies have demonstrated that heifers with excitable temperament have greater plasma cortisol concentrations and decreased conception rates after TAI compared with calm heifers [6,7,8]. Plasma cortisol concentrations in heifers, however, decrease with acclimation [9] and as the handling events of a breeding protocol progress [6]. Additionally, the human–animal relationship is recognized as a major determinant of farm animal welfare and reproductive performance, with negative interactions producing fear-mediated stress responses that inhibit gonadotropin secretion and downstream reproductive function [10,11]. The consequences of stress on reproductive efficiency in beef herds extend beyond peripheral glucocorticoid concentrations to include altered gonadotropin secretion, follicular dynamics, and oocyte competence [12]. Moreover, acclimation to human handling has been shown to improve reproductive outcomes after TAI in cows [9] and natural service breeding in heifers [13]. While positive reproductive outcomes were observed with acclimation in previous studies [9,13,14], the acclimation strategy adopted in those trials was lengthy, and the additional labor costs and time investment make these strategies unlikely to be adopted in a commercial setting.

Given the reduced cortisol concentrations observed as the handling events of the OS progressed, as reported in previous studies [6], the objectives of this study were to investigate the effects of acclimating heifers to human handling and the facility during the existing handling events of an OS protocol on temperament outcomes and pregnancy rates to TAI. We hypothesized that acclimating heifers to human handling and the facility during the handling events of the OS protocol would effectively decrease temperament excitability by TAI and thus increase the percentage of pregnant heifers following TAI when compared with non-acclimated control heifers. The rationale is that the three handling events of the 7-day CO-Synch + CIDR protocol can be intentionally repurposed as recurring opportunities for behavioral habituation rather than viewed solely as technical procedures, allowing the existing handling logistics of the OS protocol to serve a dual purpose at no additional scheduling cost relative to the protocol that the producer is already implementing.

## 2. Materials and Methods

The animals utilized in this study were handled and cared for in accordance with the procedures approved by the Kansas State University Institutional Animal Care and Use Committee (IACUC; protocol #4858).

### 2.1. Animals and Temperament Evaluation

This experiment took place over 2 consecutive spring breeding seasons in 2023 (Year 1) and 2024 (Year 2). A total of 622 commercial *Bos taurus* yearling beef heifers were enrolled across five locations: heifers from all five locations were enrolled in Year 1 (4 locations in KS, 1 in ND), and three of these same five locations (all in KS) were re-enrolled in Year 2 (the remaining two were unavailable to participate in Year 2), yielding eight herd observations in total (Table 1). All heifers were commercial *Bos taurus* crossbred replacement heifers, with predominant genetic background across all herds that were Angus or Angus-influenced, consistent with regional commercial practice. Most heifers were maintained on native or improved pasture with free access to mineral supplementation, with hay and/or distillers’ grains supplemented during winter and pre-breeding months consistent with each operation’s standard management. A minority of cooperating locations housed heifers in dry-lot conditions, where feeding programs were managed according to each operation’s standard practice. All locations had earthen-floor handling facilities and holding pens. Ten days before enrollment in the OS protocol (day −10), all heifers were evaluated for reproductive tract score (RTS), chute score (CS), and exit velocity (EV). Reproductive tract scores were assessed using a 5-point scoring system based on uterine horn tone and diameter and presence of palpable ovarian structures, where 1 = immature, <20 mm diameter, no tone, no palpable structures; 2 = 20–25 mm diameter, no tone; 3 = 25–30 mm diameter, slight tone; 4 = 30 mm diameter, good tone; and 5 = > 30 mm diameter, good tone, corpus luteum present (adapted from [15,16]). Heifers with an immature tract and no palpable structures (RTS of 1) were excluded from the study, according to cooperating producers’ decision on culling heifers assigned an RTS 1. Accordingly, heifers with RTS ≥ 2 were retained and enrolled in the experiment due to the collaborating producers’ decision. Treatments were stratified by RTS at enrollment (see below) so that the distribution of pre-breeding reproductive tract maturity was balanced across Control (CTRL) and Treatment (TRT) within each location. The methods utilized to evaluate CS have been previously described [17]. While restrained in the chute, heifers were scored based on movement and vocalization, where 1 = calm, no movement; 2 = restless movement; 3 = frequent movement and vocalization; 4 = constant movement, vocalization, and shaking of the chute; and 5 = violent, continuous struggling. To measure EV, two infrared sensors (FarmTek Inc., North Wylie, TX, USA) were placed 2 m apart, with the first placed chute-side, and the individual time to travel between 2 sensors was recorded. To calculate the final individual EV, the 2 m distance between the beams was divided by the recorded exit time in seconds [6]. Chute score and exit velocity have been validated as reliable indicators of cattle temperament, with substantial inter-observer agreement reported on a given handling day [18,19].

Before release from the chute on the day RTS was evaluated (day −10), heifers were stratified by RTS and CS to either receive acclimation prior to each OS event (days 0, 7, and 10; TRT; n = 307) or not (CTRL; n = 315) at each location. Stratification occurred chute-side while the heifer was still restrained. We utilized a pivot table to balance our treatments based on RTS and CS before releasing the animal from the chute. In addition, heifers were tagged with a colored ear tag indicating their respective treatment and then released from the chute. All CTRL and TRT heifers within each location were managed as one group throughout the experiment. Finally, body weight (BW; 7 locations) and body condition score (BCS; 6 locations) were recorded on day −10.

### 2.2. Reproductive Management

**Figure 1:**
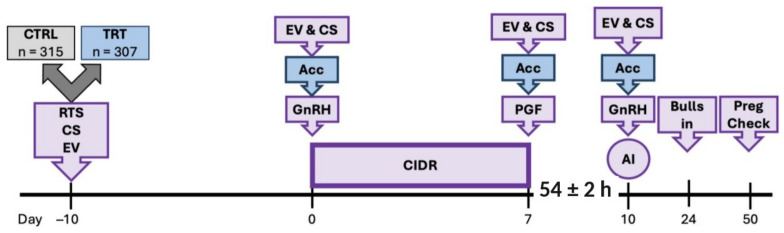
Schematic representation of the experimental design and 7-day CO-Synch + CIDR protocol. Acclimation (Acc) was applied to treatment heifers (TRT) prior to handling events on d 0, 7, and 10. All heifers were evaluated for chute score (CS) and exit velocity (EV) at each handling event. Blue boxes indicate treatment (TRT) heifers, grey boxes indicate control (CTRL) heifers, and purple boxes indicate procedures applied to all heifers.

All heifers were assigned to the 7-day CO-Synch + CIDR protocol [20,21], where they received a 100 µg injection of gonadotropin-releasing hormone (GnRH; Factrel; Zoetis Animal Health, Parsippany, NJ, USA) and a CIDR (EAZI-BREED CIDR; 1.38 g of Progesterone (P4; Zoetis Animal Health) inserted on day 0, followed by a 25 mg injection of prostaglandin F_2α_ (PGF_2α_; Lutalyse; Zoetis Animal Health) at CIDR removal on day 7. An injection of 100 µg of GnRH was administered at TAI 54 ± 2 h after CIDR removal on day 10. All heifers were exposed to bulls that previously passed their breeding soundness evaluations 7 to 15 d after TAI, according to each location’s management decisions, for the remainder of the breeding season. Transrectal ultrasonography (Ibex portable ultrasound, 5.0-MHz linear multi-frequency transducer, Ibex, E.I. Medical Imaging, Loveland, CO, USA) was performed between 40 and 91 days after TAI for pregnancy diagnosis, depending on location. At the location where diagnosis occurred at approximately 90 days post-TAI, two trained and experienced technicians distinguished TAI-conceived (approximately 90 days of gestation) from bull-bred (≤75 days of gestation, given a documented bull turn-out 7–15 days post-TAI) pregnancies based on fetal size; the difference in fetal size at these gestational ages is sufficient for reliable visual discrimination by experienced operators. Pregnancy rate to TAI was defined as the number of heifers that were diagnosed pregnant to TAI divided by the total number of heifers that were inseminated on the day of TAI. A schematic of the treatment description as well as the breeding protocol can be found in Figure 1.

### 2.3. Treatment Description

Before each breeding protocol event (days 0, 7, and 10), all heifers were brought to a holding pen near the handling facility and sorted by treatment. Treated heifers were then acclimated to human handling and the handling facility. Acclimation occurred by moving heifers from the tub, into the alley, and out through the chute without individual restraint. Following acclimation, TRT heifers were commingled with CTRL heifers, and then all heifers were brought to the handling facility for the OS protocol event for that day. The time required for the acclimation procedure was recorded at 6 locations for all three handling event days of the OS (days 0, 7, and 10). In addition, on days 0, 7, and 10, all heifers had individual CS and EV evaluated for temperament assessment (second trip for TRT heifers). On day 7, an estrus detection patch (Estrotect Breeding Indicator, Rockway Inc., Spring Valley, WI, USA) was applied to the individual heifer’s tailhead, and on day 10 was evaluated and scored based on the percentage of the patch surface that was activated as previously reported [21]. In short, patches were scored on a 5-point system, where 0 = lost patch, 1 = less than 25% activated, 2 = 26–50% activated, 3 = 51–75% activated, and 4 = more than 75% activated.

### 2.4. Blood Collection and RIA

Blood samples were collected from all heifers at one location in the spring of 2023 (n = 60; CTRL n = 30; TRT n = 30) and one location in the spring of 2024 (n = 60; CTRL n = 30; TRT n = 30). Blood was collected via coccygeal venipuncture into tubes containing sodium heparin (BD Vacutainer, Becton, Dickinson and Company, Franklin Lakes, NJ, USA) on days 0, 7, and 10, with all heifers sampled during the same handling session on each day (for TRT heifers, immediately following the acclimation pass) and stored on ice until transported to the lab within 6 h of collection for processing. Blood was centrifuged at 1500× *g* for 20 min and plasma was recovered and stored at −20 °C. Plasma cortisol concentrations were determined by solid-phase radioimmunoassay (RIA; ImmuChem Coated Tube Cortisol ^125^I kits, MP Biomedicals, LLC, Solon, OH, USA) and analyzed in duplicates. The reported intra- and inter-assay coefficients of variation (CV) were 6.1% and 9.8%, respectively. The assay sensitivity was 0.03 ng/mL. Plasma cortisol data were available for 117 of the 120 heifers originally enrolled in the cortisol sub-study; three were excluded due to sample-quality issues identified during processing.

### 2.5. Statistical Analysis

All data were analyzed using SAS 9.4 software (SAS Inst., Inc., Cary, NC, USA). Pregnancy rate to TAI was modeled with the GLIMMIX procedure assuming a binary distribution and logit link, with maximum-likelihood estimation by Laplace approximation. The model included treatment, year, estrus detection patch score, and AI technician as fixed effects, and location (5 levels) as a random effect; the random intercept on location absorbed the within-location correlation across the two years for the three locations that contributed observations in both years. As a sensitivity analysis, the same model was re-fit with location as a fixed effect and the treatment × location and treatment × year interactions tested explicitly. Repeated measures (CS, EV, and plasma cortisol concentrations) were analyzed with the MIXED procedure of SAS. Each model included treatment, year (CS and EV only), day, and treatment × day as fixed effects; location (5 levels) was included as a random effect, and within-heifer correlation across days was modeled with a compound-symmetry covariance structure on the heifer (nested within treatment) subject. The model for plasma cortisol included the fixed effect of location and excluded year, because location and year were perfectly confounded in the cortisol subset (each cortisol-sampled location was visited in only one year). Degrees of freedom were adjusted using the Satterthwaite approximation, and pairwise comparisons of least-squares means were Tukey-adjusted for multiplicity. The level of significance was set at *p* ≤ 0.05, and tendencies were determined as 0.05 < *p* ≤ 0.10. Outliers in CS and EV were identified using the standard Tukey rule (values below Q1 − 1.5 × IQR or above Q3 + 1.5 × IQR) and removed prior to analysis. One heifer with duplicate enrollment records across two locations was excluded from the repeated-measures analyses, leaving 619 heifers (2468 chute score and 2420 exit velocity observations) for those models. Pregnancy data were analyzed for the 605 heifers with complete pregnancy and patch score information.

## 3. Results and Discussion

Descriptive statistics by location are found in Table 1. Before enrollment in the OS protocol (day −10), no differences were detected between TRT and CTRL heifers in RTS (*p* = 0.78) or CS (*p* = 0.99), as was the design of the experiment. We did not use EV as a stratification criterion, because this would require all animals to be handled for a second time on day −10 to be tagged according to their treatment, and this could introduce a confounding factor with the treatment adopted herein. Nevertheless, EV (*p* = 0.87) and BCS (*p* = 0.88) for both treatment groups did not differ on day −10.

### 3.1. Reproductive Performance

**Figure 2:**
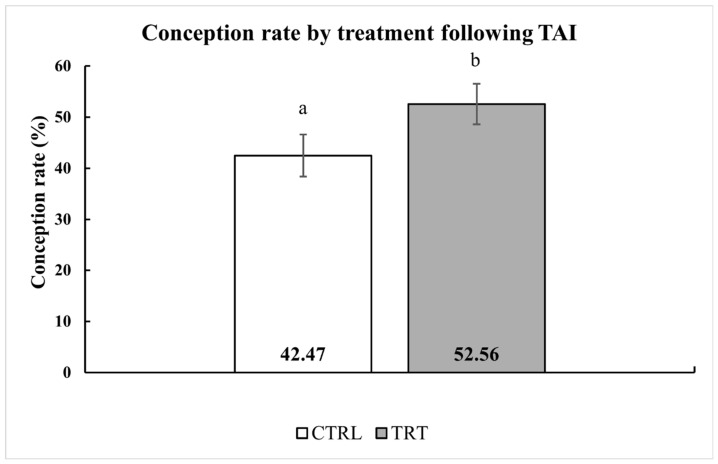
Pregnancy rates to timed artificial insemination (TAI) in acclimated (TRT) and control (CTRL) heifers. Bars with different letters differ (*p* = 0.018; LSMeans 54.5% TRT vs. 45.2% CTRL).

In the present study, acclimation to human handling and the handling facility was explored as a potential strategy to improve temperament and enhance reproductive performance in heifers. Estrus detection patch scores did not differ (*p* = 0.46) between treatments. However, acclimated heifers had greater pregnancy rates to TAI (*p* = 0.018) compared with controls (Figure 2). The treatment effect on pregnancy rate did not differ across locations (treatment × location, *p* = 0.99) or across years (treatment × year, *p* = 0.13), indicating that the acclimation benefit was consistent across the eight herd-year observations from five cooperating locations; a year main effect was detected, with overall pregnancy rates to TAI being greater in 2023 (54.7%) than in 2024 (42.8%; *p* = 0.04). Because both groups received the identical 7-day CO-Synch + CIDR protocol, the contrast between TRT and CTRL pregnancy rates isolates the additional effect of the three acclimation passes from the shared hormonal background. Results from previous studies investigating the effects of acclimation on heifer reproductive performance are mixed. While puberty was hastened in Angus x Hereford heifers that were acclimated three times weekly during a 4-week period after weaning, conception rates did not differ when insemination occurred 166 days after the final day of acclimation [22]. Additionally, puberty was hastened when Brahman-crossbred heifers were acclimated utilizing a similar method, and acclimated heifers became pregnant earlier in the breeding season than controls [13]. In that study, however, the final day of acclimation was 92 days before the start of the breeding season, and heifers were bred by natural service. These differing responses may be attributed to breed and timing of acclimation relative to breeding. Our results indicate that acclimation during the handling events of the 7-day CO-Synch + CIDR breeding protocol is associated with improved reproductive performance in *Bos taurus* yearling commercial beef heifers. Final pregnancy rates to the breeding season were not collected.

### 3.2. Temperament

**Figure 3:**
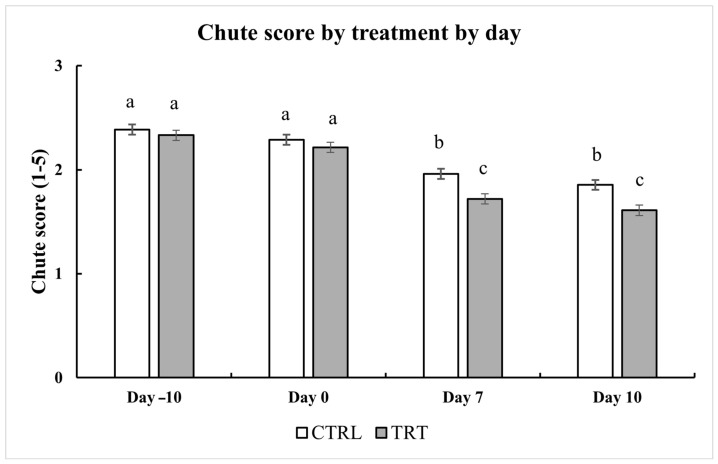
Chute scores (mean ± SEM) of heifers by treatment across handling days (days −10, 0, 7, and 10). A treatment × day interaction was detected (*p* = 0.030). Different letters indicate differences within or across days (*p* ≤ 0.05).

**Figure 4:**
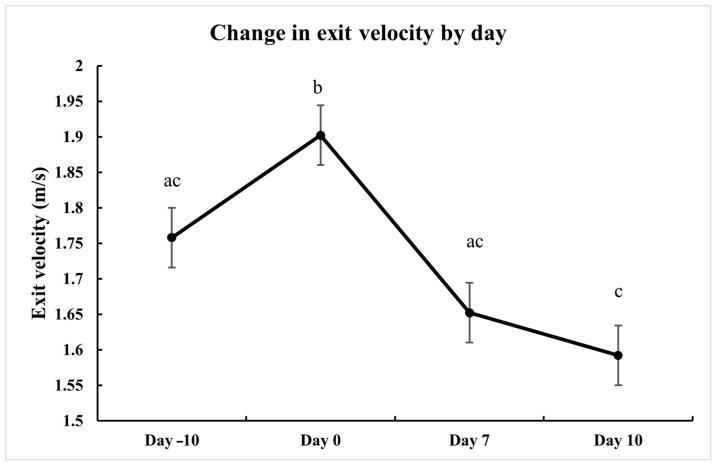
Exit velocity (mean ± SEM) of heifers across handling days. A day effect was detected (*p* < 0.0001), with no treatment effect (*p* = 0.13) or interaction (*p* = 0.91). Different letters indicate differences across days (*p* ≤ 0.05).

For the purpose of assessing temperament on a behavioral basis, we collected CS and EV for all heifers on all handling days of the experiment (days −10, 0, 7, and 10). Our results demonstrate that acclimation effectively improved CS on day 7 (*p* = 0.011, Tukey-adjusted) and day 10 (*p* = 0.010; treatment × day *p* = 0.030; Figure 3). Although acclimation failed to decrease EV (*p* = 0.13) in TRT heifers, EV for all heifers decreased from day 0 to the day of TAI (Day 10; Figure 4). A substantial year main effect was detected for EV (*p* < 0.0001), with mean EV higher in 2024 than in 2023, likely reflecting differences in environmental conditions and handling-facility configurations across the cooperating ranches contributing data each year. Directionally, the lower EV observed in 2023 compared with 2024 is consistent with the greater overall pregnancy rate to TAI in 2023 reported in §3.1 (*p* = 0.04), in line with previous reports of an inverse relationship between excitability and conception rate in beef heifers [6,7,8]. Similarly, Brahman-crossbred heifers acclimated to the handling facility and human handling for 4 weeks post-weaning had improved CS, but EV did not differ between treatments [13]. In contrast, a 4-week post-weaning acclimation period did not produce differences in CS between acclimated and non-acclimated Angus x Hereford heifers [22], but a significant treatment x day interaction was observed, with acclimated heifers exhibiting significantly decreased EV 200 days post-weaning compared with non-acclimated heifers [22]. It is worth noting that no differences in CS, EV, RTS, or BCS were detected between TRT and CTRL groups at day −10 (*p* ≥ 0.78), so the CS difference detected at TAI on day 10 cannot be attributed to a pre-existing imbalance.

In the present study, the objectives were to investigate the effects of acclimation to human handling and the facility during the OS protocol on temperament. For this purpose, plasma cortisol concentrations were analyzed in 120 heifers from 2 locations, as cortisol concentrations are often elevated during stress responses and are correlated to temperament [23]. Despite partially improved temperament in TRT heifers, indicated by reduced CS, plasma cortisol concentrations did not differ between treatments (*p* = 0.78; day *p* = 0.31; treatment × day *p* = 0.70; Table 2). Reports in the literature indicate that acclimation effectively decreased plasma cortisol concentrations in Brahman-crossbred heifers [13] and Angus x Hereford heifers [22]. Moreover, heifers enrolled in the 7-day CO-Synch + CIDR OS protocol had decreased plasma cortisol concentrations by the day of TAI when compared with the first day of the breeding protocol, regardless of the heifer’s temperament status (excitable vs. calm [6]). These authors suggest that the handling events of the 7-day CO-Synch + CIDR OS protocol may have an acclimatory effect on *Bos taurus* heifers. Two non-mutually-exclusive interpretations are possible for the lack of treatment difference in plasma cortisol concentrations. First, the timing of blood collection in TRT heifers, during their second handling event of the day, immediately following the acclimation pass, may have transiently elevated cortisol relative to a baseline measurement, partially offsetting any reduction in handling-induced stress that acclimation might have provided. The reported half-life of cortisol in non-suckled cows is approximately 30 min [24], and the interval between the acclimation pass and blood collection at each handling event would have been less than this (the time between acclimation run and protocol run for TRT heifers was unfortunately not collected). Second, our temperament data (lower CS at days 7 and 10 in TRT heifers) and pregnancy rate data (greater in TRT) suggest that the behavioral and reproductive benefits of acclimation may operate through pathways other than peripheral cortisol concentrations on handling days—for example, changes in chronic HPA-axis tone, sympathetic nervous system reactivity at TAI, or learned reduction in fear responses to handling stimuli. Taken together, these data do not support a strict-physiological claim that acclimation “mitigated stress”; rather, they support the more limited claim that acclimation improved behavioral indicators of temperament and increased pregnancy rate to TAI without producing a measurable change in plasma cortisol on handling days. The lack of treatment difference in estrus detection patch activation is consistent with the absence of a treatment effect on plasma cortisol: had acclimation reduced cortisol-mediated suppression of GnRH and LH secretion, downstream effects on dominant follicle development and estrus expression would have been expected. Accordingly, temperament differences in cattle have been linked to underlying differences in autonomic-nervous-system tone, with temperamental cows showing higher sympathetic and lower vagal activity than calm cohorts [25]. Beyond cortisol, temperament differences in cattle are also associated with differences in innate immune function and acute-phase protein responses to stressors [26], reinforcing the view that the welfare and productivity consequences of cattle temperament operate through multiple integrated physiological systems rather than a single endocrine axis.

For producers to adopt the use of acclimation commercially, three criteria must be met: the method must be time-efficient, labor-conscious, and yield a significant positive impact on a production outcome. In the present study, the outcome of interest was pregnancy rates to TAI. Acclimation, achieved by moving heifers through the tub, alley, and chute without restraint, satisfied all three criteria. The time required for the acclimation procedure was recorded at 6 locations (n = 252) for all three handling event days of the OS. At these locations, mean acclimation time was 17 s per heifer. The longest single acclimation event recorded was 17 min and 28 s (Location 4, Day 7, n = 38 TRT heifers; Table 3), which we report here as a conservative upper bound on the time investment required at commercial scale. The procedure could realistically be implemented with no more than two experienced cattle workers in many facilities, although fewer workers available may increase the time required to implement this strategy. Finally, our acclimation procedure was replicated in different commercial locations, equipped with different handling facility design settings, and consistently yielded greater pregnancy rates to TAI (treatment × location *p* = 0.99; Table 1). The effectiveness of any acclimation strategy depends on the skill of the personnel performing it; quantitative tools such as Stockman’s Scorecard [27] can be used to evaluate and standardize handler practices in commercial settings, complementing the time- and procedure-based metrics reported here.

Several limitations of this study should be acknowledged. First, the colored ear tags used to identify TRT and CTRL heifers were visible to personnel performing AI, which could have introduced unconscious bias in semen handling, insemination technique, or restraint practices. The colored-tag approach was selected because the trial was designed to integrate into the existing handling logistics of cooperating commercial ranches without disrupting their established schedules; alternatives that would have preserved full blinding were not operationally feasible at the multi-location commercial scale of this study. Artificial insemination was performed by trained technicians following standardized procedures at each location, and AI technician was included as a fixed effect in the statistical model, which itself was not a significant predictor of pregnancy rate (F(12, 582) = 1.21, *p* = 0.271); the treatment effect on pregnancy rate to TAI was robust to inclusion of AI technician, suggesting that any technician-level bias was minimal relative to the treatment effect. Future studies conducted in research-station settings should adopt fully blinded designs. Second, the diagnostic window for pregnancy diagnosis (40–91 days post-TAI) reflects the operational constraints of the cooperating ranches; at the location where diagnosis was performed at approximately day 90 post-TAI, fetal-size differentiation between TAI-conceived and bull-bred pregnancies was performed by experienced technicians, but earlier diagnosis would have provided greater precision. Because the treatment × location interaction was non-significant (*p* = 0.99), the late-diagnosis location did not contribute disproportionately to the overall treatment effect on pregnancy rate to TAI. Third, our outcome variable is pregnancy at the diagnostic time point, which conflates true conception with any subsequent early embryonic loss (fertilization rates in beef heifers approach 90%, with the majority of embryonic loss occurring between Days 8 and 16 of gestation [28], and pre-implantation embryonic loss has been estimated at 0–13% across studies [29]; excitable cows have also been reported to experience greater pregnancy loss than calm cohorts [30]); distinguishing these mechanisms would require early diagnostic methods and frequent assessment of animals, which by itself is challenging due to the stress of handling. Fourth, plasma cortisol was analyzed in a subsample of 117 heifers (of 120 originally enrolled in the cortisol sub-study) drawn from 2 of 8 herd-year observations, selected on the basis of logistical feasibility for blood-sample transport rather than randomly; the subsample was therefore not representative of all herd-year combinations in the trial and was not pre-powered to detect small between-treatment differences in plasma cortisol. The null cortisol result is best interpreted as the absence of a large detectable effect on handling-day cortisol rather than evidence of no effect. Finally, although our study did not detect a treatment difference in plasma cortisol concentrations on handling days, this null result does not rule out treatment differences in chronic HPA-axis tone, sympathetic reactivity at TAI, or other physiological pathways that may underlie the observed behavioral and reproductive effects.

## 4. Conclusions

The present study demonstrates the effects of acclimation during the handling events of the 7-day CO-Synch + CIDR breeding protocol on temperament, plasma cortisol concentrations, and reproduction of commercial *Bos taurus* beef heifers. Acclimation, applied at each of the three handling events of the 7-day CO-Synch + CIDR protocol, improved chute score on days 7 and 10 and increased pregnancy rate to TAI relative to non-acclimated controls. Exit velocity decreased over time in both groups but did not differ between treatments, and plasma cortisol concentrations did not differ between treatments on the handling days. Together, these results indicate that the behavioral and reproductive benefits of this acclimation strategy were not mediated by detectable changes in peripheral cortisol on handling days; the underlying mechanism may instead involve changes in chronic HPA-axis tone, sympathetic reactivity at the moment of TAI, or learned reduction in fear responses to handling. The acclimation procedure was time-efficient (mean of 17 s per heifer; longest single event recorded ≤ 18 min per location-day), implementable with two cattle workers, and consistently associated with improved pregnancy rates to TAI across eight herd-year observations from five locations, supporting it as a practical strategy for beef producers seeking to improve reproductive outcomes in replacement heifers.

## Figures and Tables

**Table 1:** Characteristics of heifers by location (mean ± SEM).

Pregnancy Rate to TAI, % ^5^			RTS ^2,4^	BCS ^2,3^	BW (kg)	n	Year ^1^	Location
CTRL	TRT	Overall						
59.0	59.0	59.0	3.56 ± 0.06	5.3 ± 0.04	336.5 ± 3.1	125	1	1 ^a^
48.7	58.3	38.5	3.22 ± 0.07	5.3 ± 0.04	327.8 ± 2.8	125	2	2 ^a^
61.6	66.6	56.6	3.12 ± 0.11	6.3 ± 0.07	354.7 ± 3.7	60	1	3 ^b^
46.0	52.6	39.4	3.47 ± 0.06	6.9 ± 0.07	402.9 ± 3.3	76	2	4 ^b^
40.9	43.3	38.7	3.74 ± 0.10	6.0 ± 0.05	366.7 ± 3.8	61	1	5 ^c^
26.3	34.4	17.8	3.57 ± 0.10	NA ^6^	359.1 ± 3.4	60	2	6 ^c^
56.0	58.0	54.2	3.15 ± 0.08	6.0 ± 0.06	377.9 ± 3.4	66	1	7
51.0	54.1	48.0	4.71 ± 0.08	NA ^6^	NA ^6^	49	1	8
49.8	54.5	45.2	3.50 ± 0.03	5.8 ± 0.03	355.7 ± 1.6	622	--	**Overall**

^1^ Year 1 = Spring 2023; Year 2 = Spring 2024. ^2^ Values are reported as mean ± SEM. ^3^ BCS = Body condition score (1–9 scale). ^4^ RTS = Reproductive tract score (1–5 scale). ^5^ No treatment × location interaction was detected for pregnancy rate to TAI (*p* = 0.99). Per-location values are raw pregnancy rates within each location; the Overall row reports treatment LSMeans from the GLIMMIX model. ^6^ Data not collected in these locations due to limited personnel availability. ^a,b,c^ Indicate the same physical ranch enrolled in both years; specifically, Locations 1 and 2 (^a^), 3 and 4 (^b^), and 5 and 6 (^c^) represent three ranches that contributed data in both Year 1 and Year 2.

**Table 2:** Plasma cortisol concentrations (ng/mL; LSMean ± SE) of acclimated (TRT) and control (CTRL) heifers across handling days of the 7-day CO-Synch + CIDR protocol.

Day	CTRL	TRT
0	13.93 ± 1.64	13.60 ± 1.64
7	14.17 ± 1.62	14.59 ± 1.63
10	13.33 ± 1.64	11.94 ± 1.64
Across Days	13.81 ± 1.29	13.38 ± 1.29

Treatment *p* = 0.78; day *p* = 0.31; treatment × day *p* = 0.70. n = 117 heifers from 2 locations.

**Table 3:** Time (reported as min:s) required to complete the acclimation ^1^ procedure at each location on each day.

			Location			
	1	2	4	5	6	7
Day 0	7:16	8:17	12:18	6:40	7:36	5:38
Day 7	9:20	11:54	17:28	4:57	6:30	5:27
Day 10	8:38	8:11	7:15	6:17	7:13	4:23
n ^2^	62	62	38	30	30	30

^1^ Acclimation consisted of moving heifers through the tub, alley, and chute without restraint. ^2^ “n” indicates the number of TRT heifers at each location contributing to the time measured for applying treatment across days 0, 7, and 10. Locations 3 and 8 were not collected due to limited personnel availability.

## Data Availability

The data presented in this study are available upon reasonable request from the corresponding author. The data are not publicly available due to ongoing analyses.
